# Active hiding of social information from information-parasites

**DOI:** 10.1186/1471-2148-14-32

**Published:** 2014-03-03

**Authors:** Olli J Loukola, Toni Laaksonen, Janne-Tuomas Seppänen, Jukka T Forsman

**Affiliations:** 1 Department of Biology, University of Oulu, POB 3000, Oulu FI-90014, Finland; 2 Department of Biology, University of Turku, Turku FI-20014, Finland; 3 Department of Biological and Environmental Science, University of Jyväskylä, POB 35, Jyväskylä FI-40014, Finland

## Abstract

**Background:**

Coevolution between pairs of different kind of entities, such as providers and users of information, involves reciprocal selection pressures between them as a consequence of their ecological interaction. Pied flycatchers (*Ficedula hypoleuca*) have been shown to derive fitness benefits (larger clutches) when nesting in proximity to great tits (*Parus major*), presumably because they this way discover and obtain information about nesting sites. Tits suffer from the resulting association (smaller clutches). An arms race between the tits (information host) and the flycatchers (information parasite) could thus result. Great tits often cover eggs with nesting material before, but not during incubation. We hypothesized that one function of egg-covering could be a counter-adaptation to reduce information parasitism by pied flycatchers. We predicted that tits should bring more new hair to cover their exposed eggs when a pied flycatcher is present near to tit nest than when a neutral (non-competing) species is present. We conducted decoy and playback experiment in Oulu and Turku, Finland. First, we removed and collected all the hair covering the tit eggs. Then, we measured how the perceived presence of flycatcher or waxwing (*Bombycilla garrulus*) affects tits' egg-covering by collecting and weighing the hair brought on the eggs and photographing the nest 24 h after the playback.

**Results:**

Tits brought more hair into the nest and covered the eggs more carefully after flycatcher treatment, compared to waxwing treatment. We also found that the tits in Oulu (over 600 km to north from Turku) had more hair on the top of their eggs in general.

**Conclusions:**

Together, these results suggest that the counter-adaptation function of egg-covering against information parasites may be an extension of original function to protect eggs from low temperatures.

## Background

Coevolution is reciprocal evolutionary change in interacting species driven by natural selection, and is one of the most fundamental ecological and genetic processes that organize earth biodiversity [1,2]. The reciprocal selection pressures can occur between hosts and parasites, hosts and symbionts or predators and prey, often leading to adaptive responses [2,3]. Interspecific competition also can lead to coevolutionary dynamics, and is expected to lead to character displacement [4], niche segregation, and avoidance of competing species [5-7]. However, all interactions between competing species are not necessarily purely antagonistic. Recent studies suggest that overlap in resource use can also result in positive net effects at least to one party if the presence, behaviour or performance of one species can be used as a source of information about mutually exploited resources by putative competitors [8,9]. If such positive effects increase with increasing ecological overlap, then the species utilizing another as an information source can be under selection to converge traits with the source species [8], or to develop behavioural adaptations to increase ecological overlap dynamically [10,11]. Because increased overlap in resource use may result in costs for the information source in terms of enhanced interference and exploitation competition [12], an evolutionary arms race [13] on acquiring and hiding information could result [8].

Great tits (*Parus major*) and pied flycatchers (*Ficedula hypoleuca*) are putatively competing species sharing many aspects of their ecology, such as nest sites, predators and food [14]. Our previous studies have shown that flycatchers use tits as a source of information in habitat and nest-site selection decisions and gain fitness benefits from doing so [12,15,16]. In particular, the clutch size of tits seems to be of importance for flycatchers because they frequently visit tit nests during the nest-site selection (O. Loukola, unpublished observations) and later during the breeding period [17] despite of the mortality risk of visiting tit nests personal observation [18,19]. A potential reason for visits in tit nests is that flycatchers use tit preferences in adjusting their own choices of nest-site characteristics [20], an important niche dimension in birds [21]. The clutch size of tits is an important factor in this respect because flycatchers’ probability to copy or reject tit preference depends on whether tits have a high or low clutch size, respectively [10,11,22]. Female great tits with good problem-solving skills produce larger clutches in smaller foraging ranges and with shorter workday lengths than do non-solver females, regardless of the quality of a nesting habitat [23]. For prospecting flycatchers, the clutch size of tits may reveal the competence of the observed individual in decision-making and therefore trigger general response to copy.

Flycatchers’ propensity to be attracted to the vicinity of tit nest and copy the behavioural traits of successful (high clutch size) tits may lead to niche convergence [11] and results in increased costs for the tits. The spatial proximity of flycatchers has indeed been shown to entail fitness costs due to competition for the tits, resulting in smaller clutches and fewer nestlings of poorer condition [12]. In addition, overlap in use of nest sites with coexisting species may increase nest predation rates [21]. Therefore, an evolutionary arms race [13], with counter-adaptations by the information-host tits in response to the information parasitism by pied flycatcher, is expected [8]. The potential options for an information-source/host are to either (i) cease to provide the information by abandoning the activity altogether, (ii) close the window of profitable utilization of information e.g. by aggression or changing the time, place or ecological setting of the activity, or to (iii) attempt hiding the event observed by the information-parasite [8]. When the activity being spied upon is related to breeding, outright abandonment or change of circumstances is usually not possible, but aggression and concealment remain viable options.

The avian family Paridae (tits and chickadees) are one of a few groups of bird species that commonly cover eggs with nesting material [24]. As several tit and chickadee species through Europe, North America and Asia cover the eggs during egg laying, the egg covering seems to be a phylogenetically conserved trait in parids. The egg covering is costly in terms of time and energy and there is a lot of variation in this behaviour, both within and between populations [11,24]. How this behaviour leads to benefits sufficient to outweigh the costs, and which factors cause the variance of egg covering, are still unanswered questions. Many hypotheses have been put forward to explain the egg covering behaviour of tits but none of these has been able to fully explain this phenomenon. Eggs are usually covered only during the laying period, but not later in the season when the incubating parent leaves the nest to forage. For example, egg covering could be an adaptation against low temperatures, but the need for thermal insulation should be even more pronounced during incubation when embryogenesis has started. Egg covering could conceal the eggs from nest predators [24] or nest destroyers, such as house wrens (*Troglodytes aedon*) in Black-capped Chickadees (*Parus atricapillus*) and in Tufted Titmice (*P. bicolor*) in North America [25], but tit nests are already hidden in cavities and the light covering is unlikely to protect eggs from enemies if they enter the nest cavity. Moreover, the need to protect eggs should be more pronounced later in the season with greater expended investment and reduced opportunity for re-nesting.

Here, we explicitly test with a manipulative field experiment whether great tits enhance the egg-covering in the presence of pied flycatchers. Based on the ideas suggested by Seppänen et al. [8] and results of Loukola et al. [11], we predict that the great tits avoid giving information to pied flycatchers by increasing the effort to bring new hair to cover exposed eggs when pied flycatcher is present near to tit nest, compared to presence of a neutral (non-competing) species.

## Results

**Figure 1:**
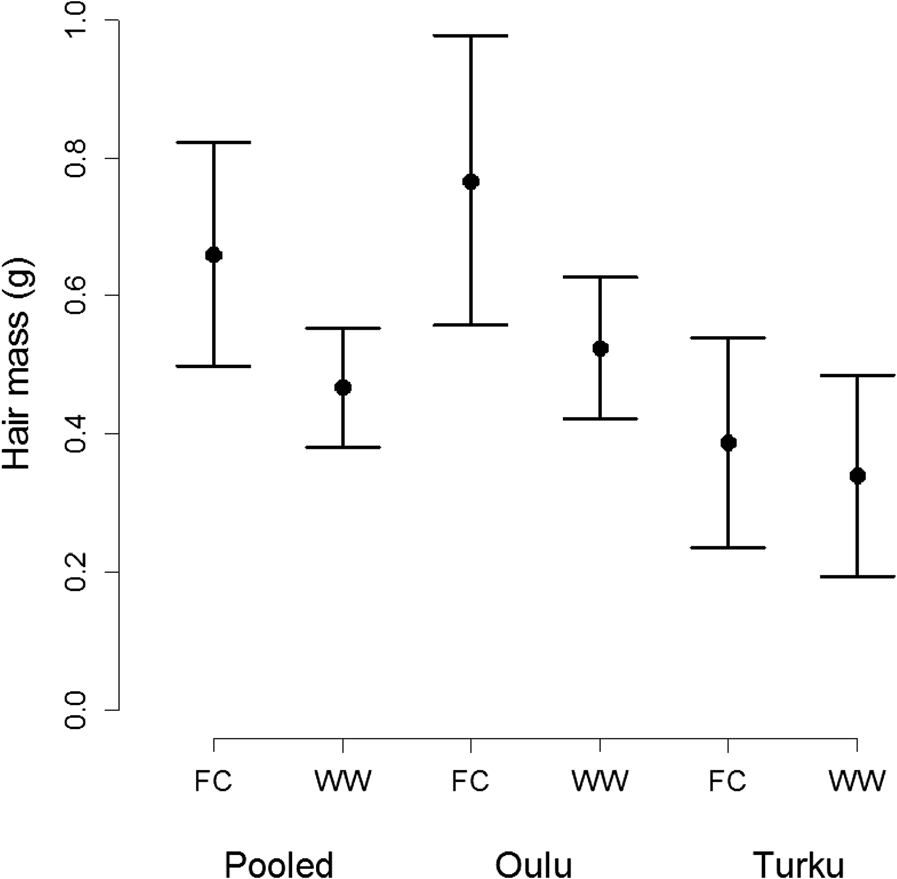
**Hair masses that great tits brought on the eggs within 24 hours after the playback treatments.** Solid dots represent the mean masses of the hair that great tits brought on the eggs after the pied flycatcher and waxwing treatments in both areas together (pooled, n = 59), Oulu (n = 41) and Turku (n = 18), respectively. Bars represent confidence interval for the hair mass at a 95% confidence level. FC = Flycatcher, WW = Waxwing.

**Figure 2:**
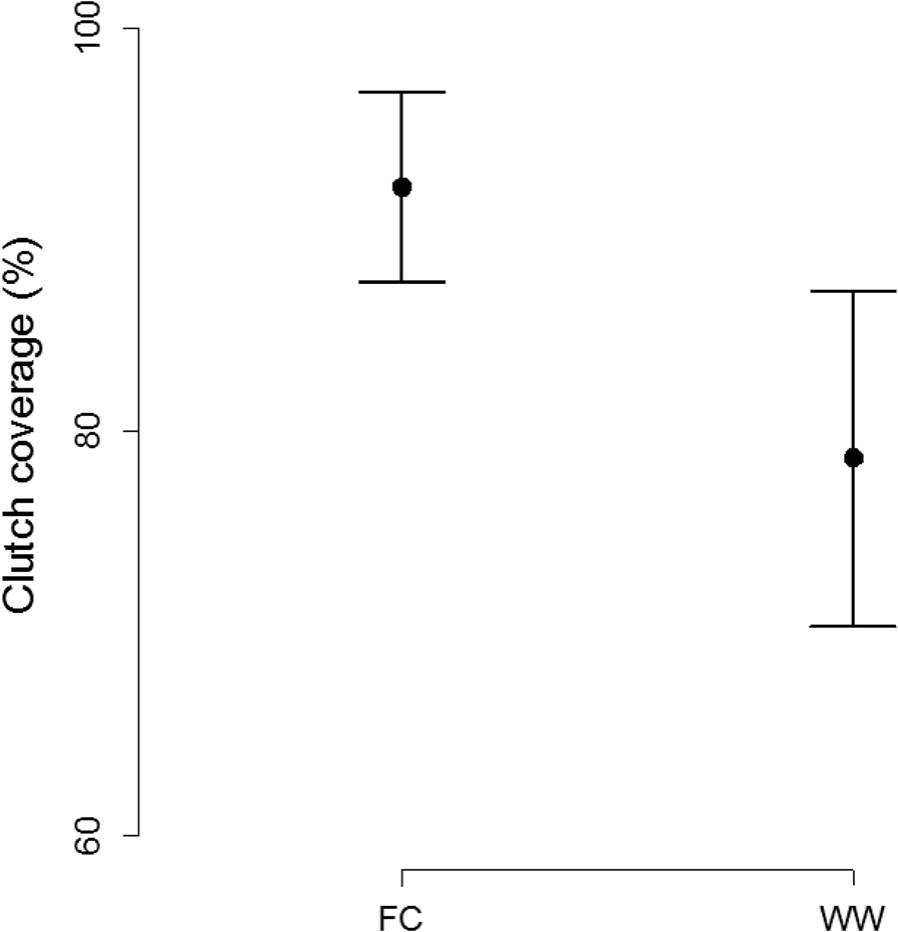
**Coverages (%) of the hair that great tits brought on the eggs 24 hours after the playback treatments.** Solid dots represent the mean coverage of the hair that great tits brought on the eggs after the pied flycatcher and waxwing treatments, respectively. Sample size is 53 (43 in Oulu and 10 in Turku). Bars represent confidence interval for the clutch coverage at a 95% confidence level. FC = Flycatcher, WW = Waxwing.

Baseline hair samples and hair samples 24 hours after the playbacks were collected from 59 tit nests (from 41 and 18 nests in Oulu and Turku, respectively). Clutch coverages were measured from 53 tit nests (from 43 and 10 nests in Oulu and Turku, respectively). All parameter estimates for the linear mixed effect models are presented in Tables 1 and 2. The mean values and 95% confidence intervals for great tits’ responses to playback treatments, hair sample mass and clutch coverage 24 hours after the playbacks, are presented in Figures 1 and 2, respectively.

**Table 1:** Results of linear mixed effect modelling (lme): effects to hair mass

**Variables**	**est.**	**Std.Error**	**DF**	**t-value**	**p-value**
(Intercept)	0.56	0.16	56	3.58	0.0007
Treatment (FC)	0.19	0.08	55	2.44	0.0179
Baseline hair mass	0.15	0.06	56	2.66	0.0103
Order (2)	-0.14	0.08	55	-1.84	0.0717
Area (Turku)	-0.20	0.11	56	-1.87	0.0674

*Note:* All of the lme results were obtained using restricted maximum likelihood (REML).

**Table 2:** Results of linear mixed effect modelling (lme): effects to clutch coverage

**Variables**	**est.**	**Std.Error**	**DF**	**t-value**	**p-value**
(Intercept)	82.20	6.51	51	13.71	0.0000
Treatment (FC)	13.75	3.64	51	3.78	0.0004
Order (2)	-5.63	3.64	51	-1.54	0.1287
Area(Turku)	-11.83	7.38	51	-1.60	0.1149

*Note:* All of the lme results were obtained using restricted maximum likelihood (REML).

We found that playback treatment (flycatcher vs. waxwing (*Bombycilla garrulus*)) affected the egg covering behaviour of great tits (Tables 1 and 2). On average, tits brought 41.23% (0.19 g) more hair on the eggs (Figure 1, Table 1) and covered the eggs 17.07% more (Figure 2, Table 2) in response to the flycatcher treatment than in response to the waxwing treatment.

The pre-experiment baseline hair sample mass, i.e. the amount of hair on the eggs before the first playback, predicted the mass of the new hair brought to the nest (Table 1). This means that tits that had a lot of hair on the eggs before the experiment also brought more hair in response to the removal of hair. Order of the playback (flycatcher first vs. second) had marginally non-significant effect to hair sample masses (Table 1).

**Figure 3:**
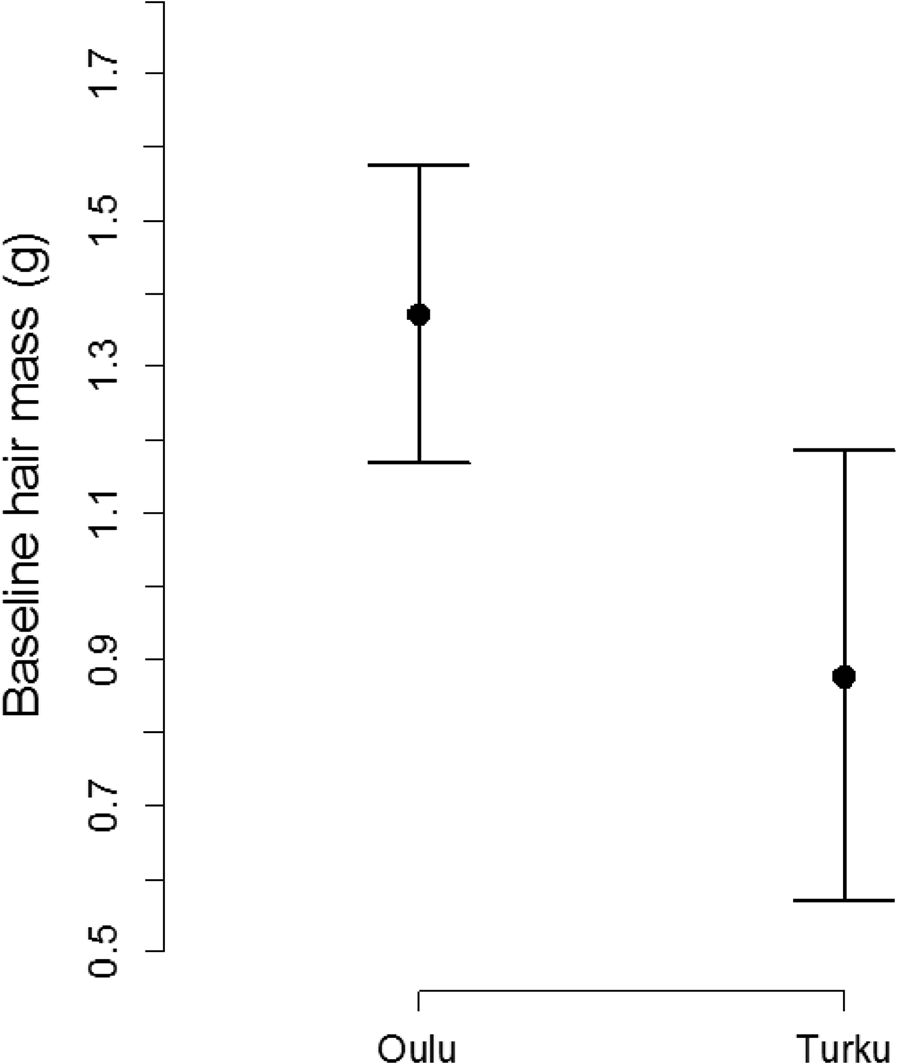
**Pre-experiment baseline hair sample masses of the hair on the great tit eggs.** Solid dots represent the mean pre-experiment baseline hair sample masses of the hair on the eggs in Oulu (n = 43) and Turku (n = 18), respectively. Bars represent confidence interval for the pre-experiment baseline hair sample mass at a 95% confidence level.

The study areas differed (but not significantly) in the hair sample masses (Figure 1, Table 1). On average, tits in Oulu brought 97.70% (0.38 g) and 54.08% (0.18 g) more hair on the eggs 24 h after flycatcher and waxwing playbacks, respectively, than did tits in Turku. There was however no significant interaction between study area and playback treatment (lme; hair mass ~ (treatment (flycatcher) + area (Turku)) ^2: F_1,55_ = 1.23, t = -1.11, p = 0.27). The study areas differed significantly in the pre-experiment baseline hair sample masses (lme; baseline hair mass ~ area (Turku): *F*_1,57_ = 4.29, *t* = -2.07, *p* = 0.04, Figure 3). Tits in Oulu had 54.50% (0.48 g) more hair on the eggs before removal experiments. This means that tits in Oulu bring more hair on their eggs in general (regardless of the treatment). We verified that this difference in baseline hair mass between the areas did not confound the difference in the response between the study areas by removing it from the model. This did not change the result qualitatively (area-effect remained significant: F_1,57_ = 7.86, t = -2.84, p = 0.01).The coverage (%) response was not different between the study sites.

## Discussion

We experimentally demonstrated that the simulated presence of flycatchers affected the egg covering behaviour of great tits in two distant study areas. Tits brought more hair on the eggs and covered them more carefully after flycatcher playback treatment compared to the treatment with a neutral species (the waxwing). These results suggest that one function of egg covering behaviour of great tits could be a counter-adaptation against information use by pied flycatchers. Our results are in line with the idea suggested by Seppänen et al. [8] that if interspecific information use entails costs for the individuals being observed, adaptations to hide the information may emerge.

The clutch size of the great tit female reveals its competence in decision making [23] and the pied flycatchers indeed use it as a primary cue of whether to copy or reject observed tit choices, such as a novel nest site feature preference [11]. By covering the eggs, tits hide this information from flycatchers. Without the information about the tit success, flycatchers may reject the behaviour of the observed tits more frequently and may be less likely to settle in the immediate neighbourhood of the tit nest. In line with this, our recent study suggested that flycatchers tended to reject the choices of ostensibly successful tits when the clutch was covered [11].

If there is an arms-race for acquiring and hiding information, do flycatchers have counter-adaptations to overcome egg-covering cf. [26]? One option is to enter the nest cavity and remove the material covering the tit eggs. Excitingly, in Oulu, pied flycatcher females have been seen to remove some hair on the tit eggs (O. Loukola, unpublished video material). Similarly, in Öland, Sweden, collared flycatcher (*Ficedula albicollis*) males have been seen to remove some of the nest material from the tit nests and great tits to bring it back after a while (D. Wheatcroft, pers. comm.). Flycatchers do not use hair in their own nests. Whether flycatchers are removing hair from tit nests in order to uncover the eggs and expose their information content warrants further studies.

Our demonstration that egg covering behaviour functions to hide information does not exclude the other potential functions of egg covering. Egg covering in different environments and populations may initially provide different direct fitness benefits also in populations where pied flycatchers arrive only after tits have already started incubating or in populations without the presence of pied flycatchers. For example, in this study, tits had more hair on the eggs before the initiation of the experiments and brought more hair within 24 hours after experiments in Oulu than in Turku, which is located over 600 km south from Oulu. The mean daily temperature from the period 1981–2010 in Oulu in May (breeding period) is 7.8°C while in Turku it is 10.2°C [27]. This supports the hypothesis suggested by Haftorn & Slagsvold [24] that the egg covering is an adaptation to low temperatures during egg-laying. They found that egg covering of great tits tended to be negatively related to the increasing ambient air temperature. Similarly, recent studies [28,29] show that the mass and insulation capacity of the nests of great and blue tits (*Cyanistes caeruleus*) are negatively correlated with the temperature. However, ambient air temperature did not affect hair mass or clutch coverage in this study. Another explanation for the differences in the pre-experiment baseline hair mass and hair sample mass between Oulu and Turku may be the differences in the egg laying synchronies of great tits and pied flycatchers in those areas. The timing of egg laying of tits and flycatchers is closer to each other in Oulu (O. Loukola, unpublished observations) than in Turku [30,31]. This means that the window of profitable information use for the flycatchers is wider in Oulu than in Turku, because great tit female leaves the nest only for short periods to feed during incubation. Hiding information may therefore have higher net value in Oulu, leading to stronger responses to flycatchers’ presence. Taken together, the pattern of geographic variation in tits' egg-covering behaviour is consistent with the geographic mosaic of coevolution theory that predicts coevolution occurs at the population scale and may result in different outcomes in different localities [2].

## Conclusions

To conclude, we show here that the presence of pied flycatcher resulted in increasing effort of great tits in bringing new hair to cover the eggs, providing evidence that species being used as information sources may develop adaptations to hide information. Co-evolution between the information users and sources in different populations may involve a variable series of adaptations and counter-adaptations leading to ever more intricate patterns of social information use.

## Methods

We conducted a field experiment in mixed and coniferous forests in Finland near the cities of Oulu and Turku in the spring 2012. The sizes of the study areas were approximately 160 km^2^ in Oulu and 3 km^2^ in Turku and contained altogether 61 separate experimental setups (43 in Oulu and 18 in Turku).

### Playback protocol and response measurements

Experiment commenced at a given tit nest when the fourth egg had been laid. The date on which the first egg was laid was ensured by checking each nest every fourth day. Experiments were done during daytime, between 10:00 and 14:00 hours.

**Figure 4:**
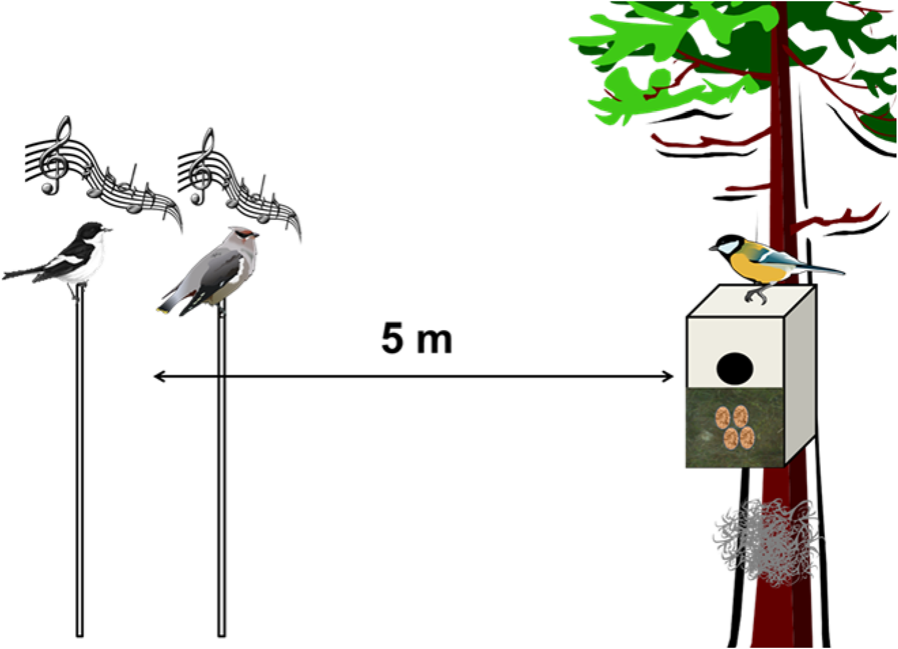
**Schematic representation of the experimental design.** First, standard amount of sheep hair was added in the vicinity of the nest. Second, when tits had four eggs in the nest photo was taken from the nest in order to get raw baseline of clutch coverage rate. Third, all the hair that covered tit eggs and nest cup was removed/collected and photo was taken from the nest. Fourth, a playback in the proximity of the tit nest was carried out. The playback consisted of either pied flycatcher song and decoy, or a song and decoy of a wax wing. Playback lasted 5 minutes. Both playbacks were carried out following the same procedure in the same nest on consecutive days in random order. Fifth, response data (hair mass and coverage %) were collected 24 hours after the end of a playback.

1. Surroundings were observed to ensure the absence of the flycatchers, after which a researcher approached the nest box.

2. The tit nest was photographed in order to get the measurement of the clutch coverage i.e. the proportion of the visible clutch surface (%).

3. All the hair that covered tit eggs and nest cup was removed to expose the eggs, and placed in a zip lock bag for later measurement and the nest was photographed again.

4. Standard amount of sheep hair (c.a. 2 grams) was attached to the tree immediately below the nest box so that material availability was similar to all trials.

5. A stuffed decoy of either pied flycatcher male or waxwing (randomly assigned) was placed in the proximity (5 metres) of the tit nest and a corresponding song playback was carried out (Figure 4). Playback lasted 5 minutes.

6. A researcher returned to the nest 24 h after the playback, collected all the hair that covered tit eggs and nest cup, and repeated the procedure steps 1 – 5, using the other decoy species (thus both treatments were applied to each tit nest, but in randomized order).

7. Response data for the second playback was measured with the same procedure, again 24 h after the playback.

Great tits were captured later when chicks were 13 days old by using passive nest box traps. Age was classified in the field and later ensured from the photographs of the wing and tail feathers, classifying individuals as one-year-old or old (at least 2 years old) [32].

Masses of the collected hair samples were weighed to the nearest 0.0001 g by using an Ohaus AS120S analytical balance. Because tits may use different covering materials (hair, moss, moss sporangia, grass etc.) with different specific gravities in different nest sites, also the proportion of the visible clutch surface (interspace between eggs was not included to surface) was measured. The clutch coverage was calculated by comparing the proportions of the visible clutch surfaces from photos taken from the nest before and after hair removals using ImageJ software (US National Institutes of health, http://imagej.nih.gov/ij). The clutch surface was measured using ‘set scale’ (the diagonal of the nest box as a known distance to apply to the image as a scale) and freehand tracing and area calculator tools. Clutch surfaces were measured twice from each photo in order to minimize measurement error and average values were used in analysis. The daily mean air temperatures in both study areas were taken from the local weather stations (data obtained from the Finnish Meteorological Institute).

### Statistical analysis

All analyses were done using R version 2.15.1 [33]. Linear mixed-effects models (function lme) [34] in the nlme package [35] was used to estimate the factors affecting the response variables, hair sample mass and clutch coverage 24 hours after the playbacks. Response variables were analysed separately. The full models included following explanatory variables: playback treatment (flycatcher vs. waxwing), order of the playback (flycatcher first vs. second), study area (Oulu vs. Turku), pre-experiment baseline hair sample mass (in the model with hair mass as a response variable only), pre-experiment baseline clutch coverage (in the model with coverage as a response variable only), ambient air temperature and the two-way interactions. In addition, as both treatments were applied to each tit nest, nest box identity was included as a random effect. The full models were compared with simpler models, and the models with the smallest corrected Akaike’s Information Criterion value [36] were used in inferences (see Additional files 1 and 2). Playback treatment and study area were retained in all models because of their ecological relevance to the question. The age data were missing for many observations and were not included in model comparisons in order to use larger data and to avoid overparameterization. The effects of ages were possible to analyse without other variables in the same models and the main effects were not statistically significant. Final model for hair sample mass included playback treatment, study area, order of the playback and pre-experiment baseline hair sample mass as fixed effects. The final model for clutch coverage included playback treatment, study area and order of the playback as fixed effects. Function lme in the nlme package was used to estimate the effect of the study area (with nest box identity as a random effect) on the pre-experiment baseline hair sample mass.

### Ethical note

Research was carried out in accordance with Finnish legislation. All experiments on great tits were done under the ringing license of JF (2975) and TL (2737) and permit from Centre for Economic Development, Transport and the Environment (ELY-centre) (VARELY/353/07.01/2012). Bird handling was done with highest possible care. Handling or sampling did not cause any lasting harm to the birds.

## Competing interests

The authors declare that they have no competing interests.

## Authors’ contributions

OJL made all the analysis and interpretation of data, drafted the manuscript and approved the final version. OJL and TL collected the data used in this study. All of the authors contributed to the conception and design of the experimental setup, revised the manuscript several times and approved the final version.

## Supplementary Material

Additional file 1 AICc values for selected linear mixed-effects models explaining hair mass. Click here for file

Additional file 2 AICc values for selected linear mixed-effects models explaining clutch coverage. Click here for file
