# Supplementary material for: Active hiding of social information from information-parasites
Source: BMC Evol Biol. 2014 Mar 3;14:32. doi: 10.1186/1471-2148-14-32 (PMC3939400; doi:10.1186/1471-2148-14-32)
Supplement: Additional file 1 — AICc values for selected linear mixed-effects models explaining hair mass. [file 1471-2148-14-32-S1.pdf]

Additional file 1. AICc values for selected linear mixed-effects models explaining hair mass.

Model (lme), fixed effects	Df	AICc	ΔAICc	Akaike weights (w_i)
**hair mass ~ treatment + area + order + baseline hair mass**	**7**	**160.17**	**0.000**	**0.36**
hair mass ~ treatment + area + baseline hair mass	6	161.33	1.15	0.20
hair mass ~ treatment + area + order + baseline hair mass + treatment: order	8	161.88	1.70	0.15
hair mass ~ treatment + area + order + baseline hair mass + temperature	8	162.43	2.26	0.12
hair mass ~ treatment + baseline hair mass	5	162.64	2.47	0.10
hair mass ~ treatment + area + order	6	164.84	4.67	0.03
hair mass ~ treatment + area	5	166.00	5.82	0.02
hair mass ~ treatment + order	5	168.70	8.53	0.01
hair mass ~ treatment	4	169.91	9.73	0.00

Notes: Nest box identity was included as a random effect in all models. Akaike weights (w_i) represent the strength of evidence in favor of model i being the best model
