# Supplementary material for: Active hiding of social information from information-parasites
Source: BMC Evol Biol. 2014 Mar 3;14:32. doi: 10.1186/1471-2148-14-32 (PMC3939400; doi:10.1186/1471-2148-14-32)
Supplement: Additional file 2 — AICc values for selected linear mixed-effects models explaining clutch coverage. [file 1471-2148-14-32-S2.pdf]

Additional file 2. AICc values for selected linear mixed-effects models explaining clutch coverage.

Model (lme), fixed effects	Df	AICc	ΔAICc	Akaike weights (w_i)
**clutch coverage ~ treatment + area + order**	**6**	**979.69**	**0**	**0.178**
clutch coverage ~ treatment + area	5	979.87	0.17	0.163
clutch coverage ~ treatment + area + order + baseline clutch coverage	7	979.98	0.28	0.154
clutch coverage ~ treatment + area + baseline clutch coverage	6	980.10	0.41	0.145
clutch coverage ~ treatment + area + order + baseline clutch coverage + treatment: area	8	980.65	0.96	0.110
clutch coverage ~ treatment + area + order + baseline clutch coverage + treatment: baseline clutch coverage	8	980.99	1.30	0.093
clutch coverage ~ treatment + area + order + treatment: order	7	981.94	2.25	0.058
clutch coverage ~ treatment + area + order + baseline clutch coverage + treatment: order	8	982.21	2.52	0.051
clutch coverage ~ treatment + area + order + baseline clutch coverage + temperature	8	982.26	2.57	0.049

Notes: Nest box identity was included as a random effect in all models. Akaike weights (w_i) represent the strength of evidence in favor of model i being the best model
